# Effectiveness of Antenatal Clinics to Deliver Intermittent Preventive Treatment and Insecticide Treated Nets for the Control of Malaria in Pregnancy in Kenya

**DOI:** 10.1371/journal.pone.0064913

**Published:** 2013-06-14

**Authors:** Jenny Hill, Stephanie Dellicour, Jane Bruce, Peter Ouma, James Smedley, Peter Otieno, Maurice Ombock, Simon Kariuki, Meghna Desai, Mary J. Hamel, Feiko O. ter Kuile, Jayne Webster

**Affiliations:** 1 Department of Clinical Sciences, Liverpool School of Tropical Medicine, Liverpool, United Kingdom; 2 Kenya Medical Research Institute/Centers for Disease Control Research and Public Health Collaboration, Kisumu, Kenya; 3 Disease Control Department, London School of Tropical Medicine and Hygiene, London, United Kingdom; 4 Centers for Disease Control and Prevention, Atlanta, Georgia, United States of America; Menzies School of Health Research, Australia

## Abstract

**Background:**

Malaria in pregnancy can have devastating consequences for mother and baby. Coverage with the WHO prevention strategy for sub-Saharan Africa of intermittent-preventive-treatment (IPTp) with two doses of sulphadoxine-pyrimethamine (SP) and insecticide-treated-nets (ITNs) in pregnancy is low. We analysed household survey data to evaluate the effectiveness of antenatal clinics (ANC) to deliver IPTp and ITNs to pregnant women in Nyando district, Kenya.

**Methods:**

We assessed the systems effectiveness of ANC to deliver IPTp and ITNs to pregnant women and the impact on low birthweight (LBW). Logistic regression was used to identify predictors of receipt of IPTp and ITN use during pregnancy.

**Results:**

Among 89% of recently pregnant women who attended ANC at least once between 4–9 months gestation, 59% reported receiving one dose of SP and 90% attended ANC again, of whom 57% received a second dose, resulting in a cumulative effectiveness for IPTp of 27%, most of whom used an ITN (96%). Overall ITN use was 89%, and ANC the main source (76%). Women were less likely to receive IPTp if they had low malaria knowledge (0.26, 95% CI 0.08–0.83), had a child who had died (OR 0.36, 95% CI 0.14–0.95), or if they first attended ANC late (OR 0.20, 95% CI 0.06–0.67). Women who experienced side effects to SP (OR 0.18, CI 0.03–0.90) or had low malaria knowledge (OR 0.78, 95% CI 0.11–5.43) were less likely to receive IPTp by directly observed therapy. Ineffective delivery of IPTp reduced its potential impact by 231 LBW cases averted (95% CI 64–359) per 10,000 pregnant women.

**Conclusion:**

IPTp presents greater challenges to deliver through ANC than ITNs in this setting. The reduction in public health impact on LBW resulting from ineffective delivery of IPTp is estimated to be substantial. Urgent efforts are required to improve service delivery of this important intervention.

## Background

Malaria in pregnancy can have devastating consequences for both mother and baby, yet the harmful effects are preventable [1]. In 2007, approximately 32 million pregnancies occurred in malaria-endemic areas in sub-Saharan Africa [2]. The World Health Organisation’s (WHO) recommended strategy for malaria prevention and control during pregnancy in areas of stable malaria transmission in Africa is a package of intermittent preventive treatment (IPTp) with two doses of sulphadoxine–pyrimethamine (SP), regular use of insecticide treated nets (ITNs), and effective management of clinical malaria and anaemia. These interventions are commonly delivered through antenatal clinics (ANC) through a collaboration between malaria and reproductive health programmes [3]. IPTp and ITNs can substantially reduce disease burden and adverse outcomes of malaria in pregnancy [4]–[7], and are inexpensive and cost effective [5], [6], [8]. The Roll Back Malaria Partnership (RBM) aims to ensure that 100% pregnant women receive IPTp and at least 80% of people at risk from malaria (including pregnant women) use ITNs in areas of moderate to high-intensity transmission by 2010 [9], with even more ambitious targets of 100% for both interventions by 2015 [10] and a call for universal ITN coverage [11].

In spite of almost universally high levels of ANC coverage in countries of sub-Saharan Africa (SSA) [12], coverage with the WHO recommended two doses of SP and ITNs in the majority of countries is extremely low. According to a recent review of national survey data in 27 countries with survey data between the years 2009–2011, median coverage of two doses of SP was 24.5% (range 7.3–69.4%) even though the median ANC coverage for at least two visits was 84.6% (range 49.7–96.9%, 22 countries, 2003–2011) [13] representing substantial missed opportunities at ANC. Despite all 45 malaria endemic countries having a policy of providing ITNs to pregnant women, the median use of an ITN the previous night among pregnant women in 37 countries with survey data between the years 2009–2011 was 35.3%, range 5.2–75.5 [13].

The RBM and other global health targets such as the Millennium Development Goals will not be met unless programme managers can measure, identify, and address the current barriers to the delivery of IPTp and ITNs. It is a particular concern that programmes have been unable to deliver IPTp effectively [14], [15], as this relatively simple and cost effective regimen will most likely be replaced with more complicated and expensive drug regimens or new strategies [16] in the near future due to increasing resistance to SP [5],[17]. These alternative prevention strategies will present even greater challenges for effective delivery or systems effectiveness [18], [19].

Whilst the number of studies that have explored the determinants of the uptake of IPTp and ITNs among pregnant women and/or providers has increased substantially in recent years [20], few have attempted to measure the effectiveness of the delivery system or to quantify and compare the relative impact of missed opportunities at ANC 21–23. A better understanding of the relative impact of the different factors on the delivery of these important interventions will help policy makers achieve the best value for money when considering which barriers to target in efforts to improve uptake. This study measured the effectiveness of implementation of the national policy to deliver both IPTp and ITNs to pregnant women through ANC in one district in Kenya, including the effect on the frequency and timing of ANC visits, and assessed the predictors of effective delivery.

## Methods

### Study Area

This household survey was conducted as part of a larger study to identify and quantify the major barriers to the scale up and use of interventions to control malaria in pregnancy at the district, facility, and community level. The study was conducted in Greater Nyando District (now divided into Nyando, Muhoroni and Nyakach districts) in Nyanza Province, Kenya, between February and March 2010. Greater Nyando District has a population of 355,800 (1999 census), with more than 90% living in rural areas. Malaria is perennial holo-endemic with a parasite prevalence of 8.3% among women of child bearing age (2008 unpublished data, KEMRI/CDC), peaking between April to June and October to December. HIV prevalence among women aged 15–49 years is higher in Nyanza Province compared to all other provinces in the country, 18% compared to national average of 9% [24].

Greater Nyando had a total of 40 health facilities of which 60% (24) were owned by government, 13% (5) by missions, 18% (7) privately owned and 10% (4) community run. The government facilities comprised 1 hospital, 2 sub-district hospitals, 6 health centres and 15 dispensaries, the mission owned 1 hospital, 3 health centres and 1 dispensary and the community owned 4 dispensaries.

In line with WHO recommendations on focussed antenatal care (FANC), national policy in Kenya is for pregnant women to receive a package of interventions through antenatal care at each of 4 recommended ANC visits. Malaria in pregnancy interventions are delivered alongside prevention and control of mother to child transmission of HIV (PMTCT), tuberculosis, syphilis/sexually transmitted infections, palpations and birth planning. Malaria services include a free long-lasting insecticide treated net (LLIN) provided to all women at their first ANC visit, a minimum of two doses of SP taken under DOT and prompt diagnosis and treatment of malaria episodes with an effective antimalarial alongside health education at ANC [25]. According to current national guidelines, the first dose of SP should be given to all women after quickening and subsequent dose(s) taken at least 4 weeks (one month) apart. HIV positive pregnant women taking daily cotrimoxazole should not be given IPTp-SP [25], [26].

Kenya adopted IPTp policy in 1999, followed by the delivery of ITNs to pregnant women through ANC in 2001. National level DHS data available at the time the study was designed (DHS 2003) showed that 84% of women made two or more ANC visits, 4% pregnant women received two doses of SP during ANC visits and 4% of pregnant women used an ITN the night before the survey [24]. The IPTp and ITN coverage figures for Nyanza Province were marginally higher than the national average, with 5% reporting having received two doses of IPTp and 9% pregnant women reporting having used an ITN the night before the survey.

### Study Design and Selection of Study Participants

A two stage cluster sampling household survey was undertaken over 3 weeks during a period of intense rains, between February and March 2010. Enumeration areas from the 1999 census were used to select 40 villages, representing clusters, using probability proportional to size. A sample size of 338 women of child-bearing age (15–49 years) was sufficient to estimate an IPTp or ITN uptake of 50%, with a 6% precision, design effect of 1.75 and 10% non-response. The sample size was increased to 1,120 to maintain precision in a range of intermediate processes in the delivery of IPTp and ITNs with decreasing sample size, where women are lost from the evaluable sample at different points [27], and uses the hypothetical assumptions that 60% of women attending ANC are offered an ITN, of which 50% then use the ITN.

Households in selected clusters were mapped using Global Positioning System (GPS) software (CDC GPS Sample for Windows Mobile devices https://sites.google.com/a/wolkon.com/gps-sample/). Thirty five households were randomly selected from GPS coordinates of all households within each cluster to get 28 households with women of childbearing age as resident. One respondent within each household was selected using a set of predefined criteria as follows: women must be aged 15–49 years; pregnant women were prioritised over mothers of children aged under one year, who were prioritised over other eligible women; and if more than one eligible woman was currently pregnant/mother of a child aged under 1, the woman most closely related to the head of the household was interviewed (i.e. wife> daughter> sister> niece/aunt> daughter-in-law).

### Data Collection Instrument

The questionnaire sought to obtain key coverage indicators on the frequency and timing of ANC attendance, the frequency, timing and source of IPTp-SP doses and whether each dose was taken under DOT, and whether an ITN was obtained, by source, and used during the current/most recent pregnancy; to understand women’s knowledge of malaria in pregnancy in relation to use of IPTp and ITNs; and to determine whether HIV status alters behaviour patterns. A composite malaria knowledge score was created based on the cumulative score of correct responses in relation to source of malaria (mosquitoes), consequences of malaria in pregnancy to mother (anaemia) and unborn child (miscarriage, low birth weight, premature birth, stillbirth) and of methods to prevent malaria in pregnancy (e.g. IPTp, nets and/or ITNs). HIV status was self-reported, and additional indirect questions about recognition and use of cotrimoxazole and history of episodes of opportunistic infections associated with HIV were included. Self-reported IPTp, ITN and ANC practices, including HIV testing and results, were compared with data contained in ANC cards where available. Recording of household assets to develop a wealth index and observation of household ITNs *in situ* was also performed. Principal components analysis was used to construct a wealth index to assess socio-economic status (SES) [28], [29] using household characteristics such as education of the household head, number of sleeping rooms, floor materials, source of drinking water, type of toilet facilities, household ownership and a range of household assets.

The questionnaire was translated and back translated to ensure accuracy of translation of concepts and variables, and then pre-tested. Interviewers were trained to conduct the interviews in the two most commonly spoken local languages, Dholuo and Kiswahili.

### Analysis Framework

**Figure 1:**
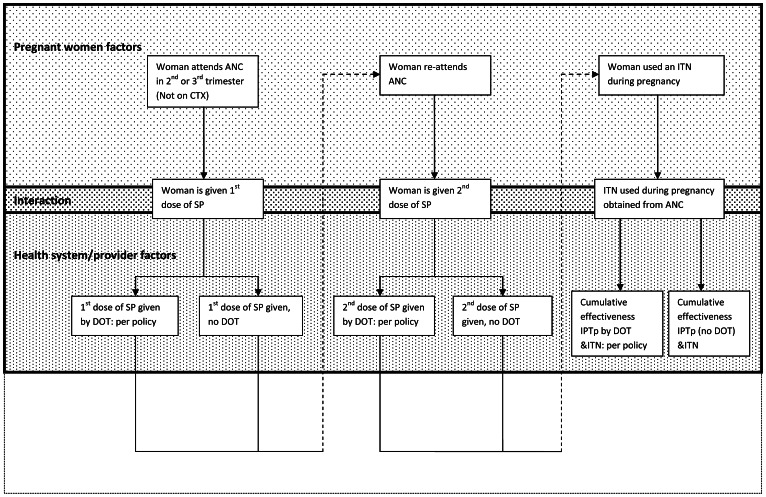
Algorithm for cumulative systems effectiveness of ANC, IPTp and ITN use. ANC: antenatal clinic; CTX: cotrimoxazole; SP: sulphadoxine-pyrimethamine; DOT: directly observed therapy; IPTp: intermittent preventive treatment; ITN: insecticide treated net.

The concept of intervention effectiveness as described by Vlassoff and Tanner showed how the translation of the efficacy of a disease control tool into community effectiveness was dependent on coverage, diagnostic accuracy and the compliance of users and providers [30]. More recently this concept has been adapted for delivery of interventions through the health system [21], and used as a health systems effectiveness framework by the malERA consultative group [33]. We adapted this concept specifically for the delivery of IPTp and ITNs through ANC to measure delivery system effectiveness using household data to identify critical points in the delivery system that were ineffective (Figure 1). We then explored the predictors of effectiveness at critical points of weakness identified in the delivery pathway.

### Data Processing and Analysis

Data from the household surveys were collected on personal digital assistants (PDAs). All analyses were adjusted for the survey design, clustering of households and sample weights using STATA 11.

Socio-demographic characteristics of pregnant and recently pregnant women were described and compared. Proportions of women accessing ANC, receiving each dose of SP by source and the timing of each dose, and receiving ITNs by source were quantified among both groups of women. The indicator for ITN use among pregnant women was the proportion of pregnant women who reported having used an ITN the night before the survey [34], [35], and for recently pregnant women the indicator used was the proportion of women who reported having used an ITN regularly during the most recent pregnancy [36], [37].

**Figure 2:**
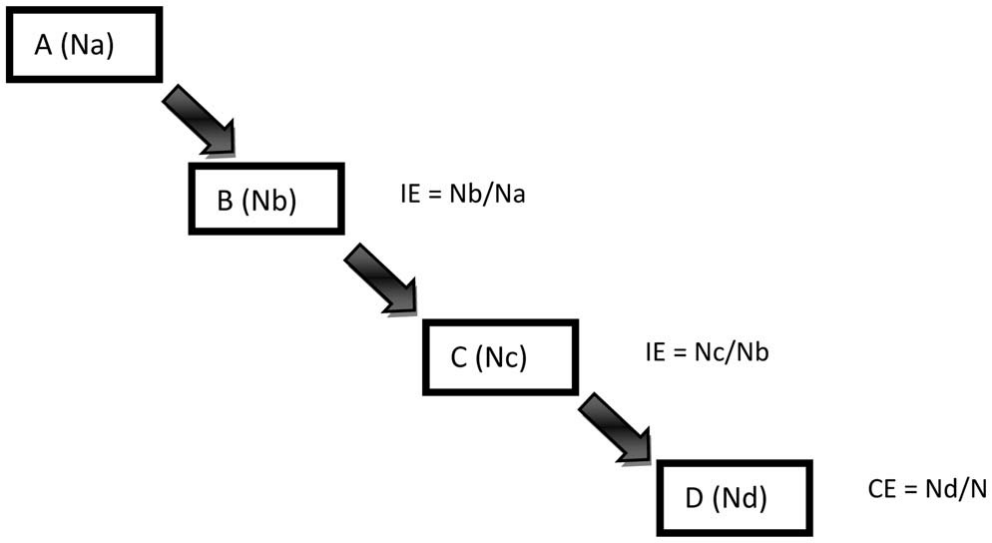
Formula for calculating intermediate and cumulative systems effectiveness. Where: N = Total number of target population; Nx = Number of women successfully completing step x IE: Intermediate effectiveness; CE: Cumulative effectiveness.

The systems effectiveness analysis was undertaken using recently pregnant women and excluded HIV positive women with documented use of cotrimoxazole. Systems effectiveness was defined as the proportion of women attending ANC who reported having received at least two doses of SP and used an ITN regularly during the most recent pregnancy. Two categories of effectiveness analyses were applied - ‘intermediate process effectiveness’ and ‘cumulative systems effectiveness’ [38]. Intermediate process effectiveness represents coverage of women at each of the intermediate steps in the delivery of IPTp, with the addition of ‘used any ITN’ and then ‘used an ITN from ANC’ together with taking the second dose of IPTp as the final process. Intermediate process effectiveness was calculated as the number of women who successfully completed the intermediate process (the numerator) as a proportion of all women who reached that point in the delivery system (the denominator) (see formula in Figure 2). An intermediate process was classified as ineffective if <80% women successfully completed that step. Cumulative systems effectiveness represents successful coverage of women for each of the intermediate steps up to that point in the delivery system. The final step represents the percent of eligible women in the community that received both interventions and is equivalent to a cumulative coverage indicator for several indicators (two ANC visits, two doses of SP (IPTp) and ITN use) that are measured by DHS.

Intermediate process effectiveness and cumulative systems effectiveness analyses for IPTp were performed on women receiving IPTp in an eligible month of gestation (4 to 9 months) and excluded women who did not receive IPTp and who were taking cotrimoxazole as documented on ANC cards as per national policy. This analysis was conducted under two scenarios, Scenario A where the criteria of receiving IPTp by DOT was not included, and Scenario B where women received IPTp by DOT i.e. per policy. The systems effectiveness analysis for ITNs was not restricted by gestational age as policy states ITNs are to be delivered to women at their first ANC visit regardless of gestation.

Univariate analyses of potential predictors of receipt of: i) two doses of SP (with or without DOT); ii) one dose by DOT; and iii) two doses by DOT (as the three least effective processes identified in the effectiveness analysis), was conducted using logistic regression models accounting for survey design, and adjusted Wald tests were applied to test for associations between predictors and outcomes. We also explored the predictors of ITN use. Predictors explored included those known to be important from previous studies, including: selected individual and household socio-demographic factors (women’s age, marital status, education, malaria knowledge using the composite malaria knowledge score, urban: rural residence and SES); gravidity; having lost a live born child; number and timing of ANC visits; whether they recently had an episode of malaria and whether they took medicine for that episode. Variance inflation factors were examined for all predictors included in the models to assess potential collinearity. Potential predictors significant at the 10% level (p<0.1) in the univariate analyses were included in multivariable logistic regression models to determine which factors were associated with the outcomes after accounting for other potential predictors. Interactions between paired predictors were assessed for all predictors included in the models.

We estimated the potential and actual effectiveness of the malaria in pregnancy prevention strategy on low birth weight (LBW) extrapolated to the pregnant population of Nyanza Province (n = 225,001). We used the protective efficacy of IPTp on LBW of 29% observed in a meta-analysis of trials which compared 2-dose IPTp with SP to case management or placebo in women during their first or second pregnancy (RR 0.71; 95% CI 0.55–0.92) [5], a prevalence of LBW of 12% (unpublished data from Siaya District Hospital, Ouma personal communication), and co-coverage with IPTp by DOT and ITNs, using the following formula [39]:
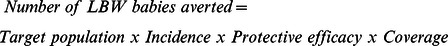



### Ethical Considerations

The study was approved by the ethical committees of the Kenya Medical Research Institute (KEMRI) National Ethics Review Committee, the Liverpool School of Tropical Medicine, the London School of Hygiene and Tropical Medicine, and the US Centers for Disease Control and Prevention in Atlanta. Written informed consent was obtained from women prior to being interviewed at home and for adolescents, both parental permission and assent of the subject was obtained.

## Results

### Study Population

A total of 961 women of childbearing age were interviewed (98% response rate) of which 121 (13%) were pregnant and 216 (23%) were mothers of children under one year, i.e. women who had been pregnant in the previous 12 months, termed ‘recently pregnant women’. The majority of women in both groups had attended primary school and were married, and the predominant ethnic group was Luo. Of the 121 pregnant women, a third were in their first trimester and the mean gestational age was 5 months (IQR 3–7) (Table 1). The median age among pregnant women was 25 years (IQR 22–30) and the majority of these women were multigravidae, with 88% of women having had 3 or more previous pregnancies and a median of 4 births (IQR 2–5), and 11% were in their first pregnancy. Five percent of women already had a child aged less than 1 year and a third had lost a live born child.

**Table 1:** Socio-demographic characteristics of the respondents.

Socio-demographic data	Pregnant women (N = 121)		Recently pregnant women (N = 216)		P value
Characteristic	n	% (95% CI)	n	% (95% CI)	
**Trimester**					
1^st^ trimester	37	30.6			
2^nd^ trimester	47	38.8			
3^rd^ trimester	37	30.6			
Median months gestation	121	5 (IQR 3–7)			
**Gravidity**					<0.0001
Gravida 1	13	10.7	31	14.4	
Gravida 2	2	1.7	50	23.3	
Gravida 3>	106	87.6	134	62.3	
Median number of births	121	4 (IQR 2–5)	215	3 (IQR 2–5)	
**Age group (15**–**49)**					0.1
15to19yr	14	11.6	28	13.0	
20to24yr	38	31.4	54	25.0	
25to29yr	38	31.4	51	23.6	
30to34yr	20	16.5	40	18.5	
35to39yr	7	5.8	31	14.3	
40+yr	4	3.3	12	5.6	
Median Age		25 (IQR 22–30)		27 (IQR 21–32)	
**Marital status**					0.8
Married	102	84.3	179	82.9	
Widowed	8	6.6	12	5.6	
Single	11	11.57	25	11.6	
**Residence**					0.7
Rural	114	94.2	201	93.1	
Urban	7	5.8	15	6.9	
**Education level**					0.05
None/nursery	3	2.5	5	2.3	
Primary	105	86.8	163	75.5	
Secondary	12	9.9	46	21.3	
College/University	1	0.8	2	0.9	
**Ethnic Group**					0.4
Luo	112	92.6	203	94.0	
Luyha	5	4.1	6	2.8	
Kalenjin	1	0.8	0	0	
Kisii	1	0.8	4	1.9	
Other	2	1.7	3	1.4	
**HIV status (self reported)**	6/46	13.0 (5.91,26.37)	8/162	4.9 (2.14,10.97)	0.07
**Women with child aged <1 year**	5/107*	4.7 (2.00, 10.4)	216/216	100.0	
**Women with child aged <5 years**	81/107*	75.7(65.5,83.6)	216/216	100.0	
**Woman has lost a child**	34/108*	31.5 (23.0, 41.3)	54/216	25.0 (19.4,31.6)	0.5

* missing records.

Of the 216 recently pregnant women, almost two-thirds had 3 or more previous pregnancies, with a median of 3 births (IQR 2–5), and a quarter of women had lost a live born child. About half the women were aged between 20–29 years, with a median age of 27 years (IQR 21–32). The only characteristics that were significantly different between the two groups of women was education (p = 0.05), with twice as many recently pregnant women having attended secondary education, and gravidity (p<0.0001).

### Coverage Indicators

#### Access to ANC and timing of first ANC visit

Among recently pregnant women, 87% of women reported attending ANC at least once, 78% at least twice and 34% made 4 or more visits, with the median number of ANC visits of 3 (IQR 2–4) (Table 2). Attendance among pregnant women was lower, with a median number of 2 visits (IQR 1–3), as would be anticipated given that 70% of women were still in their first or second trimester. The median month gestation at first ANC visit was 5 months in both groups of women (IQR 3–6 and 4–6 among pregnant and recently pregnant women respectively).

**Table 2:** Key coverage indicators for ANC attendance and malaria in pregnancy interventions among pregnant women and recently pregnant women.

Coverage Indicators		Pregnant		Recently pregnant	
		women (N = 121)		women (N = 216)	
		n	% (95% CI)	n	% (95% CI)
***Number ANC visits***					
At least one visit		59	48.8 (39.3, 58.3)	188/215*	87.4(83.0,90.9)
At least two visits		38	31.4 (23.1, 41.1)	167/215	77.7(70.9,83.2)
4 or more visits		6	5.0 (2.2, 10.6)	72/215	33.5(27.5,40.1)
Median number of ANC visits		59	2 (IQR 1–3)	188/215	3 (IQR 2–4)
Median gestation at 1st ANC visit		58	5 (IQR 3–6)	187/215	5 (IQR 4–6)
***1 dose SP***					
1 dose (all trimesters, any source)				114/216	52.8(45.9,59.5)
1 dose (all trimesters, any source)†				114/207	55.1(47.9,62.0)
1 dose (4–9 months, any source)				97/184	52.7(44.9,60.4)
Dose 1 at ANC (all trimesters)				104/114	91.2(85.5,94.8)
Dose 1 by DOT at ANC (all trimesters)				79/103	76.7(65.0,85.4)
***2 doses SP***					
2 doses (all trimesters, any source)				62/216	28.7(23.9,34.0)
2 doses (all trimesters, any source)†				62/206	30.1(25.2,35.6)
2 doses (4–9 months, any source)				51/184	27.7(22.3,33.8)
Dose 2 at ANC (all trimesters)				61/62	98.4(88.6,99.8)
Dose 2 by DOT at ANC (all trimesters)				32/59	54.2(40.6,67.2)
***3 doses SP***					
3> doses (all trimesters, any source)				18/216	8.3 (5.3, 12.9)
3> doses (all trimesters, any source)†				18/203	8.9 (5.6,13.7)
***ITN use***					
ITN used last night		97/121	80.2 (72.5, 86.1)		
ITN used during last pregnancy				192/215	89.3(83.9,93.0)
ITN sourced from ANC clinic		72/97	74.2 (64.7, 81.9)	145/192	75.5(67.6,82.0)
Co-coverage IPTp* and ITNs					
**1 dose SP**	No iptp1, No itn			14	6.8 (3.8, 11.9)
	Iptp1, No itn			9	4.4 (2.2, 8.5)
	No iptp1, itn			78	37.9(32.2,43.9)
	Both iptp1 & itn			105	51.0(43.8,58.1)
**2 doses SP**	No iptp2, No itn			21	10.2 (6.5, 15.7)
	Iptp2, No itn			2	1.0(0.2, 3.9)
	No iptp2, itn			122	59.5(53.4,65.3)
	Both iptp2 & itn			60	29.3(24.2,34.9)

IPTp1: one dose of SP; IPTp2: two doses of SP; DOT: directly observed treatment; ITN: insecticide treated net; ANC: antenatal clinic.

† Excludes women who did not receive IPTp and who were taking cotrimoxazole as documented on ANC cards.

#### Number of IPTp doses and timing of receipt

The coverage analysis shows that just over half of recently pregnant women reported receiving one dose of SP in any trimester from any source (55%), and 53% if women who received doses in the first trimester were excluded (Table 2). The majority of women who reported receiving the first dose of SP at ANC said they received it under DOT. Only 30% of women reported receiving the recommended second dose of SP (any trimester from any source); 28% in an eligible trimester. The majority of women reported receiving the second dose from ANC and just over half reported receiving it by DOT. A small proportion of women (9%) reported receiving three doses of SP.

#### IPTp coverage by number and timing of ANC visits

**Figure 3:**
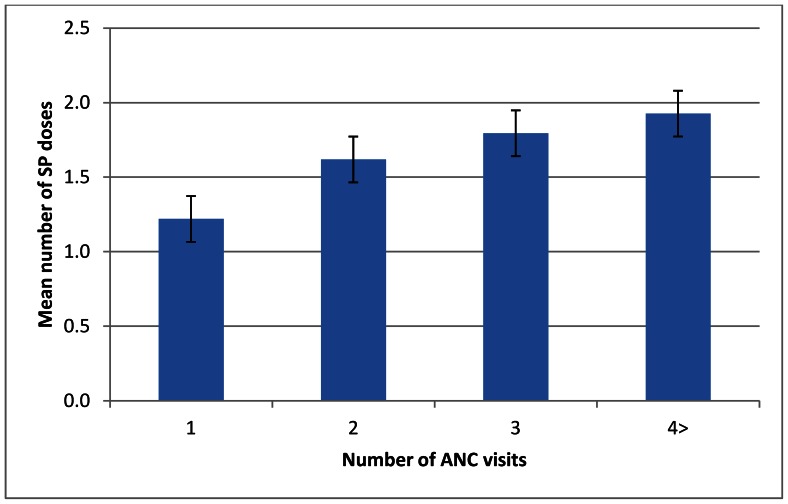
Mean number of SP doses received by number of ANC visits among recently pregnant women.

**Figure 4:**
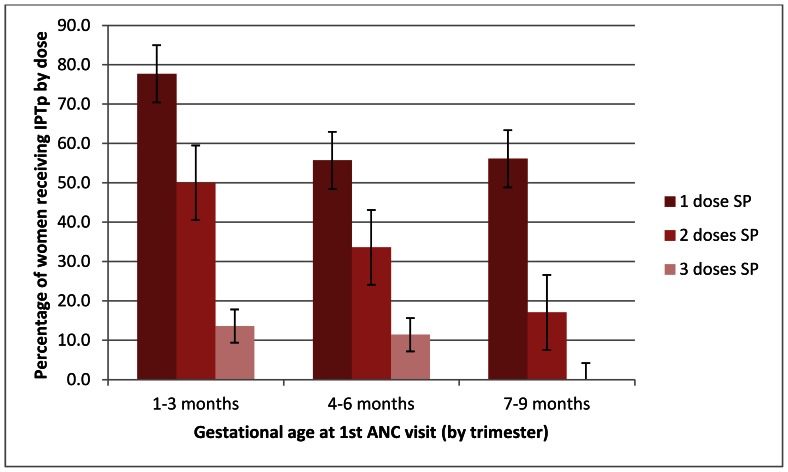
Number of SP doses received by trimester at 1^st^ ANC visit among recently pregnant women.

Among recently pregnant women, the mean number of doses of SP showed a clear trend with increasing number of ANC visits (p = 0.03) (Figure 3). Similarly, the number of doses of SP received was associated with gestational age at first ANC visit, with fewer recently pregnant women receiving a second dose of SP if they first attended ANC in their second or third trimesters (p = 0.03) (Figure 4).

#### ITN use and co-coverage with ITNs and IPTp

Among pregnant women, 80%reported using an ITN the previous night whereas 89% of recently pregnant women reported using an ITN regularly during their most recent pregnancy. Approximately three quarters of women in both groups reported obtaining the net used in pregnancy from ANC. In terms of co-coverage of ITN use and IPTp receipt among recently pregnant women, 51% reported using an ITN and receiving one dose of SP and 29% of using an ITN and receiving two doses of SP. Whilst few women reported using neither ITN nor one or two doses of SP, a large proportion of women reported using an ITN alone in the absence of any SP.

### Systems Effectiveness of IPTp and ITN Delivery through ANC

#### IPTp with or without DOT

Although 89% (164/185) of recently pregnant women visited ANC at least once in an eligible trimester, only 59% received the first dose of SP, and of the 90% of women who returned for a second ANC visit, only 57% received a second dose (Table 3). Only 23% (42/185) of women received the recommended two doses of SP and used an ITN during pregnancy. There were two intermediate processes in the delivery system that were <80% effective - the receipt of one and two doses of SP. ITN use among women receiving two doses of SP was 90%.

**Table 3:** Intermediate process and cumulative effectiveness of receiving two doses of SP and using an ITN with and without DOT in an eligible gestation (4–9 months) among recently pregnant women.

	2 doses of SP				2 doses of SP by DOT			
Intermediate process	N	n	Individual delivery process effectiveness % (95% CI)	Cumulative delivery effectiveness %	N	n	Individual delivery process effectiveness % (95% CI)	Cumulative delivery effectiveness %
**Attend ANC at least once**	185	164	88.6 (83.7, 92.2)	88.6	185	164	88.6 (83.7, 92.2)	88.6
**Took IPTp1**	164	96	58.5 (50.8, 65.9)	51.9	164	96	58.5 (50.8, 65.9)	51.9
**Took IPTp1 by DOT**					89*	67	75.3 (63.9, 84.0)	36.2
**Attend ANC at least twice**	96	86	89.6 (81.6, 94.3)	46.5	67	60	89.6 (78.4, 95.3)	32.4
**Took IPTp2**	86	49	57.0 (46.8, 66.6)	26.5	60	35	58.3 (44.7, 70.8)	18.9
**Took IPTp2 by DOT**					34*	25	73.5 (55.7, 86.0)	13.5
**Took IPTp2 & used an ITN during last pregnancy**	49	47	95.9 (84.6, 99.0)	25.4				
**Took IPTp2 & used an ** ***ANC*** ** ITN during last pregnancy**	47	42	89.4 (77.7, 95.3)	22.7				
**Took IPTp2 by DOT & used an** ***ANC*** ** ITN during last**					25	25	100.0	13.5

IPTp1: one dose of SP; IPTp2: two doses of SP; DOT: directly observed treatment; ITN: insecticide treated net; ANC: antenatal clinic.

* missing records.

#### IPTp given by DOT

**Figure 5:**
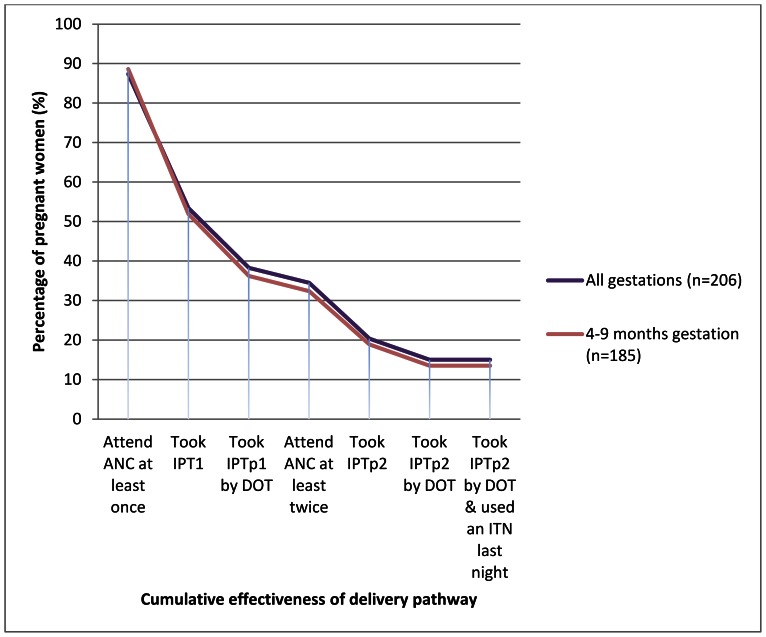
Cumulative effectiveness of ANC to deliver IPTp and ITNs among recently pregnant women.

With respect to DOT, the cumulative system effectiveness was even lower, with only 14% (25/185) of women receiving two doses of SP by DOT and using an ITN (Table 3; Figure 5). The intermediate processes that were <80% effective were, in addition to receipt of one dose of SP (as above), receiving it under DOT, the receipt of a second dose of SP, and receiving it under DOT. ITN use among women receiving two doses of SP by DOT was 100%.

#### ITNs

Among the 87% (188/215) of recently pregnant women who attended ANC at least once, 90% reported using an ITN during their last pregnancy of which 80% were obtained from ANC (Table 4). Among the 49% (59/121) of pregnant women who attended ANC at least once, 85% reported using an ITN the previous night, of which 82% were sourced from ANC. The difference between the percentage of women who attended ANC at least once and the percentage of women who attended ANC but were not using an ANC ITN was 25% among recently pregnant women and 15% among pregnant women.

**Table 4:** Intermediate process and cumulative effectiveness of receiving an ITN from ANC among pregnant women and recently pregnant women.

	Pregnant women				Recently pregnant women			
Intermediate process	N	n	Individual delivery process effectiveness% (95% CI)	Cumulative delivery effectiveness %	N	n	Individual delivery process effectiveness % (95% CI)	Cumulative delivery effectiveness %
**1. Attend ANC at least once**	121	59	48.8 (39.3, 58.5)	48.8	215	188	87.4 (83.0, 90.9)	87.4
**2. Used an ITN during last pregnancy/last night**	59	50	84.7 (71.2, 92.6)	41.3	188	169	89.9 (84.5, 93.5)	78.6
**3. Used an ** ***ANC*** ** ITN during last pregnancy/last night**	50	41	82.0 (64.9, 91.8)	33.9	169	135	79.9 (71.9, 86.1)	62.8
**Difference in coverage between** **process 1 and 3**				14.9				24.6

ITN: insecticide treated net; ANC: antenatal clinic.

### Predictors of IPTp Receipt

#### Two doses of SP

The univariate predictors of recently pregnant women reporting having received two doses of SP were timing of ANC visits, not having a child who died prior to this pregnancy, and malaria knowledge (Table 5). After adjustment, all three factors were independently associated with receipt of two doses of SP among recently pregnant women (Table 5). Receipt of two doses of SP progressively decreased the later a woman made her first ANC visit (OR 0.20, 95% CI 0.06–0.67 for 7–9 months gestation). Women were less likely to receive two doses of SP if they had lost a live born child (OR 0.36, 95% CI 0.14–0.95), and decreased progressively with lower knowledge of malaria (OR 0.26, 95% CI 0.08–0.83 for the lowest score).

**Table 5:** Univariate and multivariate analysis of predictors of receiving *two doses of IPTp* among recently pregnant women.

	Recently pregnant women (n = 206)							
Determinant	n	%	Crude OR	95% CI	P value	Adjusted OR	95% CI	P value
***Timing of 1st ANC visit by trimester***					0.04			0.04
**0**–**3 months**	21	52.4	1			1		
**4**–**6 months**	125	35.2	0.49	0.17,1.46		0.49	0.16,1.47	
**7**–**9 months**	38	18.4	0.21	0.06,0.68		0.20	0.06,0.67	
***Child died***					0.01			0.04
**No**	157	35.0	1			1		
**Yes**	49	14.3	0.31	0.13,0.75		0.36	0.14,0.95	
***Malaria knowledge score***					0.01			0.04
**Score 3 (Highest)**	113	38.9	1			1		
**Score 2**	59	22.0	0.44	0.21,0.94		0.47	0.21,1.09	
**Score 1 (Lowest)**	34	14.7	0.27	0.10,0.73		0.26	0.08,0.83	

#### One and two doses of SP by DOT

The univariate predictors of recently pregnant women reporting having received one dose of SP under DOT were SES and not having an unpleasant side effect after taking the dose, and for two doses of SP by DOT, malaria knowledge score and not having an unpleasant side effect after taking the previous dose of SP (Table 6). After adjustment for other potential predictors, low SES and having an unpleasant side effect after taking the dose remained significantly associated with receipt of one dose of SP under DOT. Women from the richest households were more likely to receive one dose of SP by DOT than women from the poorest households (OR 11.30, 95% CI 1.44–88.57). Women reporting an unpleasant side effect after taking the first dose of SP such as nausea, vomiting or itchy skin were less likely to receive the first dose of SP (OR 0.30, 95% CI 0.11–0.81).Previous experience of having an unpleasant side effect after the first dose of SP (OR 0.18, 95% CI 0.03–0.90) and lower knowledge of malaria (OR 0.78, 95% CI 1.11–5.43 for the lowest score) were independently associated with receipt of two doses of SP under DOT.

**Table 6:** Univariate and multivariate analysis of predictors of receiving one and two doses of SP *under DOT* among recently pregnant women.

	One dose SP under DOT (N = 103)								Two doses SP under DOT (N = 57)							
Determinant	n	%	Crude OR	95% CI	P value	AdjustedOR	95%CI	P value	n	%	Crude OR	95%CI	Pvalue	Adjusted OR	95%CI	Pvalue
***Malaria knowledge score***					0.31								0.08			0.02
**Score 3 (Highest)**	64	78.1	1						39	51.3	1			1		
**Score 2**	26	80.8	1.18	0.34, 4.02					13	84.6	5.23	0.93,29.51		7.57	1.43,40.05	
**Score 1 (Lowest)**	13	61.5	0.45	0.15, 1.38					5	40.0	0.63	0.09,4.66		0.78	0.11,5.43	
***Socio-economic status***					0.07			0.05					0.93			
**1 most poor**	19	52.6	1			1			9	44.4	1					
**2**	21	71.4	2.25	0.54, 9.13		2.61	0.63,10.84		12	58.3	1.75	0.23, 13.35				
**3**	17	94.1	14.40	1.78,116.70		12.35	1.85,82.48		9	66.7	2.50	0.32, 19.36				
**4**	25	76.0	2.85	0.71,11.46		2.48	0.61, 10.11		16	62.5	2.08	0.31, 14.05				
**5 least poor**	21	90.5	8.55	1.23,59.55		11.30	1.44,88.57		11	54.6	1.5	0.27, 8.21				
***Side effect from 1st dose***					0.02			0.02					0.05			0.04
**No**	74	82.4	1			1			40	67.5	1			1		
**Yes**	29	62.1	0.35	0.14,0.85		0.30	0.11,0.81		15	33.3	0.24	0.06,1.01		0.18	0.03,0.90	

### Predictors of ITN Use

The univariate predictors of recently pregnant women not having used an ITN were person:net ratio and a low malaria knowledge score. Person:net ratio was also a univariate predictor among recently pregnant women for not using an ITN and women taking antimalarials for a malaria episode during her most recent pregnancy were more likely to use an ITN (Table 7). After adjustment there were no independent predictors for ITN use in either group.

**Table 7:** Univariate and multivariate analysis of predictors of *using an ITN* among pregnant women and recently pregnant women.

	Pregnant women (n = 121)								Recently pregnant women (n = 215)							
Determinant	n	%	CrudeOR	95%CI	Pvalue	AdjustedOR	95%CI	Pvalue	n	%	CrudeOR	95%CI	P value	Adjusted OR	95%CI	Pvalue
***Took medicine for malaria***					NS								0.007			0.6
**No**									13	69.2	1			1		
**Yes**									99	93.9	6.89	1.76,27.00		1.77	0.15,20.31	
***Malaria knowledge score***					0.05			0.90					NS			
**Score 3 (Highest)**	49	87.8	1			1										
**Score 2**	36	77.8	0.49	0.20, 1.17		0.86	0.15, 4.83									
**Score 1 (Lowest)**	36	72.2	0.36	0.16, 0.81		0.66	0.11, 4.00									
***Person : Net ratio***					0.07			0.06					0.01			0.2
**<2**	22	95.4	1			1			22	100	na			na		
**2**–**<4**	48	100	na			na			119	99.2	1			1		
**4+**	36	72.2	0.12	0.01,1.19		0.12	0.01,1.10		53	86.8	0.06	0.01,0.48		0.21	0.02,2.40	

na–not available.

### Effectiveness of the IPTp strategy

The potential effectiveness of the malaria in pregnancy prevention strategy among the pregnant population of Nyanza Province attending ANC, which assumes that recently pregnant women not taking cotrimoxazole who made one visit in an eligible trimester (89%; 164/185) and returned to ANC for a second visit (90%; 60/67) (Table 3) received two doses of SP by DOT and used an ITN, is 6,264 cases of LBW averted. Based on the reported co-coverage of two doses of SP by DOT and ITNs among women attending ANC clinic in Nyanza Province (14%; 25/185), actual effectiveness was only 1,057 cases of LBW averted. The difference between potential and actual effectiveness is 5,207 cases of LBW (95% CI 1,436–8,080) that may have been averted if all women who attended ANC at least twice in Nyanza Province had received IPTp by DOT and used an ITN, equivalent to 231 (95% CI 64–359) cases of LBW per 10,000 women.

## Discussion

Systems effectiveness analysis was applied to household survey data to identify critical processes in the delivery of interventions to pregnant women through ANC that are least effective, providing useful data to inform strategies to strengthen service delivery in Nyanza Province. This analytical approach may be applied to national survey or other household survey data to identify weak processes in the delivery system for IPTp and ITNs at district or regional level, and their determinants, to inform regional adjustments to national strategies. However, it should be noted that national DHS surveys currently overestimate true IPTp coverage by the inclusion of women receiving SP in any trimester, and underestimate coverage in areas of high HIV prevalence where HIV-positive women are taking cotrimoxazole.

Consistent with the 2008/09 DHS survey [40], over three quarters of women had made two or more ANC visits at the time of our survey (DHS, 84%), and the median gestation at first ANC visit was 5 months (DHS, 5.7 months). In other words, although women typically start their ANC visits late in the second trimester of pregnancy, a high proportion attended twice, with each visit in an eligible gestation to receive SP. However, less than a third of women who attended ANC at least twice during their most recent pregnancy received the minimum of two doses of SP as recommended by national policy, although this figure is likely to be an underestimate due to low documented use of cotrimoxazole (14% compared to HIV seroprevalence among pregnant women participating in a previous study of 21%) [41]. This figure is higher than the DHS 2008–09 survey (17.2% for Nyanza Province), which would be anticipated given the ongoing scale-up of the national programme in the interim period of over one year. Furthermore, the DHS denominator is women who received IPTp during the pregnancy for their last live birth in the two years preceding the survey.

Based on the coverage analysis presented in Table 2, the pattern of ANC attendance among individual women could not be ruled out as a possible reason for low coverage with two doses of SP, - for example women not returning for a second ANC visit. The intermediate process effectiveness analysis revealed that the most ineffective processes occurred at the health facility. The high proportion of women who attended ANC in an eligible gestation of pregnancy and received their first dose of SP who then made a second ANC visit (90%) demonstrates that women’s ANC attendance was not the primary cause for the ineffectiveness of the IPTp strategy observed. Consistent with other studies, our findings suggest that health provider practices rather than women’s ANC attendance are primarily responsible for the ineffectiveness of the IPTp strategy in this setting [23], [42]–[44]. The two predominant reasons given by women in our study for not taking the second dose of SP were that they were not informed a second dose was necessary (33%; 15/45) and that they were not offered it (31%, 14/45) (data not shown). The finding that health workers do not always offer IPTp to women at ANC has been observed in other studies [44], [45]. Nevertheless, three quarters of women said they received the dose(s) by DOT suggesting that, where IPTp is given, it is predominantly given under DOT. However, as these data are reported rather than observed practices, this finding is not conclusive on the practice of IPTp provision by health workers.

One of the three predictors of receipt of two doses of SP was timing of women’s first ANC visit, which is influenced by a number of factors including pregnancy uncertainties, particularly during the first trimester, implications of pregnancy disclosure, interactions with health providers, and the cost of ANC procedures and the compulsory nature of follow-up appointments [46]. Women who initiated ANC during their first trimester were more likely to receive two doses of SP even though they are not eligible for IPTp at the first visit, suggesting that women’s receiving the ANC screening package early increases the likelihood of receiving IPTp on subsequent visits. The timing of ANC visits by women determines the provision of IPTp by health providers according to gestational age, as recommended by policy, though the pattern of health worker behaviour is not always consistent with policy. Even women who attend ANC at the correct gestational age are not always offered IPTp [23], [44] and this has been associated with confusion among health workers from unclear guidelines. In the Tanzania study the authors estimated that adherence to simplified guidelines by health workers as recommended by WHO had the potential to increase IPTp coverage by 20% [23]. In contrast to another study [47], both our study and the Tanzania study did not identify number of ANC visits among pregnant women as an independent predictor of receiving two doses of SP, suggesting that the number of ANC visits alone is insufficient to ensure uptake in the presence of other barriers. Whilst we cannot comment on the precise nature of provider level factors based on this analysis, these factors will be explored using data collected in health facilities as part of the same study. Nevertheless, stock out of SP is unlikely to be a primary issue as stock outs would impact similarly on uptake of both one and two doses of SP.

The two other individual level factors that predicted non receipt of two doses of SP, having a child who died and malaria knowledge score, appear to be consistent with the national DHS data, which show that IPTp coverage increases with educational status and wealth quintile [40] if malaria knowledge score and child died are accepted as proxy indicators for educational status and SES respectively [48]. However, this finding has not been consistently observed in previous studies, which show substantial variation [44], [45]. SES was also a predictor of receiving the first dose of SP under DOT. The second predictor of not receiving both doses of SP under DOT was having had an unpleasant side effect after taking the first dose of SP, which has not previously been reported according to a recent systematic review [20]. This suggests that health workers are less likely to administer SP by DOT to women who complain of a previous side effect from SP.

In contrast to IPTp, ITN use among pregnant and recently pregnant women was high (>80%). While three quarters of women reported receiving their ITNs from ANC, some women used an ITN obtained from other sources. It is not possible from the data to know whether this is because these women were not offered an ITN at ANC since this was not measured. However, because most women first attended ANC in their second trimester, these women may not have been sleeping under ITN during the first trimester of pregnancy which has been identified as a high risk period associated with increased risk of LBW, miscarriage and maternal anaemia [49], [50]. Whilst not significant in the multivariate analysis, ITN use was associated with household person: net ratio which concurs with findings from another study, which showed that intra-household access to ITNs was the strongest and most consistent determinant of use among children [51].

Our analysis shows that ANC is a more effective delivery channel for ITNs than for IPTp in this setting. Whilst this paper does not present an analysis of the data collected at health facilities, data from other studies discussed here suggest that stock outs of SP, absence of cups and clean drinking water for taking SP by DOT [52], unclear guidelines [23] or confusion among health workers regarding the guidelines [53], [54], and other related health facility factors hamper delivery of IPTp. It will be important to evaluate the recent WHO IPTp policy update, which aims to simplify guidance by recommending the administration of SP to pregnant women at every scheduled antenatal visit [55] on the basis of a recent meta-analysis showing that three or more doses of SP are more effective than two doses [56], to see whether this leads to an improvement in IPTp uptake. By comparison, ITNs require a single point of contact with a pregnant woman to secure ITN uptake, with additional efforts in promotion and education to ensure women use ITNs from early pregnancy.

The reduced public health impact resulting from the ineffective delivery of IPTp observed in the study was substantial. Nevertheless, our estimate is likely to be an overestimate given that the protective efficacy of IPTp in third gravidae (or more) women is less than in primi- and secundi-gravidae, is lower in women sleeping under ITNs, and that the efficacy estimate used was observed under trial conditions [5]. This may to some extent be counterbalanced by the fact that some of the women who did not receive IPTp by DOT may have taken an effective dose.

The study had several limitations. Reporting bias among women interviewed, who may feel obliged to under- or over-report access and usage of interventions for a variety of reasons, is a weakness of household survey data. To assess potential bias we measured concordance between reported and documented information using women’s ANC cards where available. We found moderate concordance between reported and ANC card data on mean number of ANC visits (Kappa score 0.585), and fair concordance between reported data on receipt of two doses of SP and use of an ANC ITN with ANC card data on provision of two doses of SP and an ITN (Kappa score 0.399 and 0.26 respectively) [57]. These results should be treated with the caveat that relatively few ANC cards were available and information on IPTp was available for only 26% of women. There was also potential selection bias among recently pregnant women; by selecting women with a child aged under one year, women who had had a pregnancy loss (miscarriage or stillbirth) may have been excluded from the recently pregnant group of women, and these women may have had different health seeking behaviours. Also, the diminishing numbers in the evaluative sample in the effectiveness analyses mean the regression analyses have less statistical power than anticipated. Lastly, the study does not evaluate why health care providers failed to deliver IPTp to eligible women; this aspect of the study is to be published separately.

The study highlights an important area for future research, to develop programme friendly tools and methods to measure delivery system effectiveness on an ongoing basis to guide local decision making by programme managers. It will also be important to evaluate the recently updated WHO policy recommendation on IPTp with SP, a simplified recommendation to give IPTp-SP to pregnant women at each scheduled antenatal care visit released in an effort to improve coverage (see http://www.who.int/entity/malaria/iptp_sp_updated_policy_recommendation_en_102012.pdf). In the future, it will be important to include evidence on delivery system effectiveness for new drug regimens which replace SP for IPTp, or new strategies such as intermittent screening and treatment, both of which will be more complex to deliver. Lastly, efforts to increase ITN use in early pregnancy should also be encouraged, perhaps through a combination of encouraging earlier first ANC attendance and provision of ITNs to women of childbearing age.

### Conclusion

In conclusion, we have used household survey data to evaluate the effectiveness of ANC to deliver ITNs and IPTp to pregnant women in Nyanza Province in western Kenya, and to identify which steps in the delivery system are least effective. Our findings show that women’s timing of ANC attendance, malaria knowledge, and having lost a live born child are important predictors of uptake of two doses of SP. However, most women attend ANC with adequate frequency in their second and third trimesters to receive a minimum of two doses of SP. Missed opportunities at health facilities appear to account for the greatest losses in strategy effectiveness, pointing to the need for programme managers to have access to diagnostic management tools that can detect bottlenecks in service delivery in a timely manner. This represents a considerable lost opportunity to improve the health outcomes in mothers and children with two of the most cost effective health interventions available in sub-Saharan Africa, with consequent economic savings to households and health services.
